# Different Hemodynamic Responses of the Primary Motor Cortex Accompanying Eccentric and Concentric Movements: A Functional NIRS Study

**DOI:** 10.3390/brainsci8050075

**Published:** 2018-04-24

**Authors:** Lénaic Borot, Grégoire Vergotte, Stéphane Perrey

**Affiliations:** EuroMov, University Montpellier, 34 090 Montpellier, France; lenaic47@gmail.com (L.B.); gregoire.vergotte@gmail.com (G.V.)

**Keywords:** elbow flexor muscle, muscle contraction, motor control, near-infrared spectroscopy, unimanual task

## Abstract

The literature contains limited evidence on how our brains control eccentric movement. A higher activation is expected in the contralateral motor cortex (M1) but consensus has not yet been reached. Therefore, the present study aimed to compare patterns of M1 activation between eccentric and concentric movements. Nine healthy participants performed in a randomized order three sets of five repetitions of eccentric or concentric movement with the dominant elbow flexors over a range of motion of 60° at two velocities (30°/s and 60°/s). The tests were carried out using a Biodex isokinetic dynamometer with the forearm supported in the horizontal plane. The peak torque values were not significantly different between concentric and eccentric movements (*p* = 0.42). Hemodynamic responses of the contralateral and ipsilateral M1 were measured with a near-infrared spectroscopy system (Oxymon MkIII, Artinis). A higher contralateral M1 activity was found during eccentric movements (*p* = 0.04, η² = 0.47) and at the velocity of 30°/s (*p* = 0.039, η² = 0.48). These preliminary findings indicate a specific control mechanism in the contralateral M1 to produce eccentric muscle actions at the angular velocities investigated, although the role of other brain areas in the motor control network cannot be excluded.

## 1. Introduction

The movement occurring when a muscle exerts tension while lengthening (e.g., sitting, running) is known as eccentric (ECC) muscle action. ECC movement as compared to concentric (CON, muscle shortening) movement is acknowledged for being characterized by high force generation at low energy expenditure [1,2]. Lower oxygen consumption of ECC than CON movement at the same workload will be explained by lower muscle activity (EMG) of agonist and antagonist muscles during ECC than CON dynamic task [3]. How ECC movement affects brain function, in particular cortical activity has not been extensively described [4,5]. However, to what extent the cortical regions in the motor network are activated during ECC muscle actions may be critical for understanding the underlying control mechanism of certain movements encountered in daily tasks (e.g., walking downstairs).

Recent advances in brain-imaging methods (electroencephalography, EEG and functional near infrared spectroscopy, fNIRS) facilitate the investigation of brain activity during movement. But only a few neuroimaging studies (for a brief review, [5]) have explored cortical activation differences between distal joint ECC and CON movements of the upper extremities. Two neuroimaging methods were used in this context, namely EEG and functional magnetic resonance imaging (fMRI). Regarding the contralateral primary motor cortex (M1), the blood-oxygen-level dependent (BOLD) signal in fMRI was found lower during ECC wrist movement [6]. This result appears in agreement with the observed reduction in corticospinal excitability obtained from transcranial magnetic stimulation technique during lengthening contractions in elbow flexors [7]. Interestingly, a reduction in the BOLD signal of the contralateral M1 has been also observed during imagined maximal ECC movement of the elbow flexors [8] but not during real ECC movement of the hand [9]. Finally, the amplitude of the major EEG-derived movement-related cortical potentials were found significantly higher during submaximal and maximal ECC movements of elbow flexors [10,11]. Overall, current evidence is inconsistent regarding the patterns of M1 activation during ECC versus CON movements due to a scarcity of studies and heterogeneous results. 

Differences in motor task parameters across previous studies (e.g., muscle mass involved, range and velocity of movement, intensity and type of lengthening contractions) and potential methodological issues (e.g., body posture and movement) of the neuroimaging methods may explain these inconsistent findings. In fMRI while the participant lies down in a supine/horizontal position, it is more difficult to actually produce maximum contractions [8] that would result in movements of the head and in turn in measurement errors during the experiment. EEG has still its limitations, since face muscles and eye movements unrelated to the task can affect the quality of recordings. fNIRS is an effective alternative method. fNIRS is an emerging non-invasive neuroimaging modality that provides continuous bedside sensitivity of the brain oxygenation state and allows measurement in upright position without major physical restraint (compared to fMRI and EEG [12]). The basic principles of fNIRS and fMRI are similar and are based on blood-oxygenation level-dependent responses, recording oxygenated (O_2_Hb) and deoxygenated (HHb) blood in the cerebral cortex [13].

For ECC movements, when resisting an imposed load (e.g., maximal isokinetic actions), the intensity of the activation signal (i.e., EMG) changes minimally in contrast to controlling the displacement of a load (as proposed in fMRI studies [6,9] with executed movement and in one EEG study [10]). In addition, to avoid the possible feedback-mediated influence of contraction velocity and muscle length across muscle action types, an isokinetic dynamometer should be used to control limb velocity during anisometric contractions. But only the EEG study of Fang et al. [11] proposed an experimental setup with maximal isokinetic actions that required participants to resist an imposed load at one slow angular velocity (30°/s), where levels of EMG activity are likely sustained over the prescribed range of motion. 

Since M1 plays a substantial role in the execution of movements, how this brain region is activated during maximal CON and ECC muscle actions needs to be further addressed in humans. Therefore, the present study was designed to compare patterns of M1 activation between ECC and CON movements of elbow flexors at low-to-moderate angular velocities. Based on the aforementioned studies, it was hypothesized that ECC movements would require higher M1 activity than CON movements when exerted at maximal voluntary effort. To test our hypothesis, we asked participants to perform maximal ECC and CON movements of elbow flexors using an isokinetic dynamometer while brain activity of the M1 regions was simultaneously recorded using fNIRS.

## 2. Materials and Methods

### 2.1. Participants

Sixteen healthy participants (12 males and 4 females, mean age 32 ± 9 years, height 1.75 ± 0.08 m and body weight 70.8 ± 11.0 kg) were recruited for this study, which was approved by the local research Ethics Committee (IRB-EM 1703A). The experiment was performed in agreement with the standards set by the declaration of Helsinki (2013) involving human subjects. Following an explanation of all procedures, risks and benefits associated with the experimental protocol, each participant gave her or his written informed consent prior to experimentation. No information was provided about the objectives of the study. None of the participants had any physiological or orthopaedic limitations that could have affected movement of the upper extremities.

Self-reported handedness was obtained with the Edinburgh Handedness Inventory [14]. All participants performed the motor tasks with their dominant arm (2 left-handed, 12 right-handed and 2 ambidextrous). The ambidextrous participants performed the motor tasks with the side they preferred when practicing sport activities involving the upper extremities.

### 2.2. Experimental Protocol

**Figure 1:**
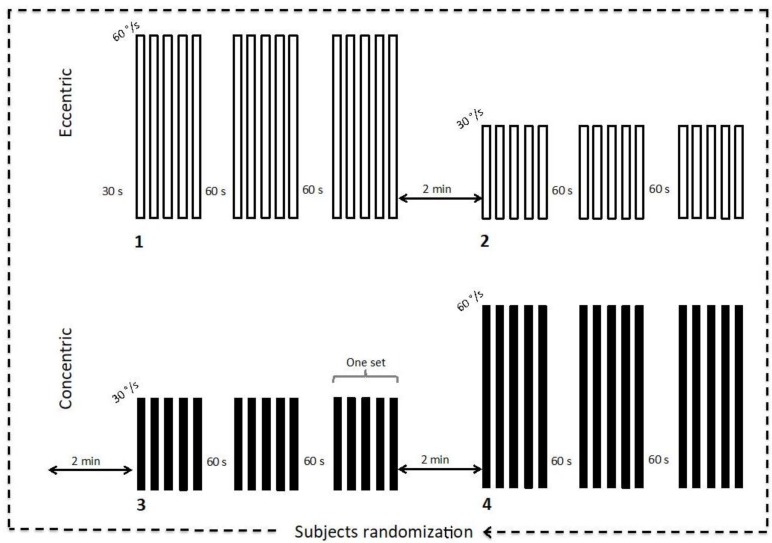
Experimental protocol. The order of the 4 experimental conditions was randomized and three sets of 5 consecutive movements were performed by condition (i.e., eccentric and concentric at angular velocities of 30°/s and 60°/s).

Participants reported to the laboratory on two separate occasions. During the first visit, participants performed familiarization trials with all materials and detailed verbal instructions on the testing protocol were provided. During this first visit, participants completed ten repetitions of ECC and CON movements with elbow flexors, beginning at a low effort and gradually increasing to efforts of maximal intensity. Following the familiarization visit (at least 24 h apart), participants were randomly assigned to perform each of the four experimental conditions to avoid order effect: ECC or CON movement for the dominant arm at two angular velocities (30°/s or 60°/s). The contralateral arm remained relaxed in a comfortable position with the hands on the thigh. Following a 30-s rest period, each experimental condition consisted of three sets of five consecutive movements with a 60-s rest interval (Figure 1) between sets [15]. Participants were instructed to contract maximally over the complete range of motion and then allow the dynamometer to passively return the limb to the starting position before beginning the next contraction. Each experimental condition was separated by 2-min rest intervals.

**Figure 2:**
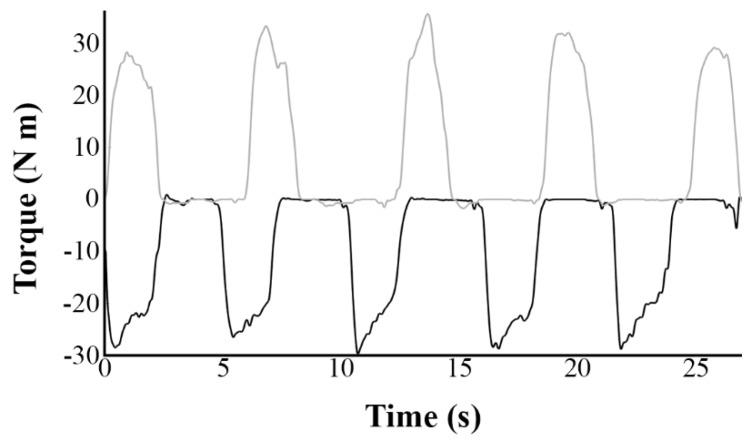
Representative recordings of torque values over five complete eccentric (black line) and concentric (grey line) movements for one participant. Anisometric contractions were performed at a constant angular velocity of 30°/s from time 0.

All movements of the dominant elbow flexors were performed on a Biodex isokinetic dynamometer (System 3, Biodex Inc., Shirley, NY, USA) in the sitting position while the back being supported and with the *trunk* and *pelvis* stabilized by using straps. Torque (N m), velocity (°/s) and position data were collected from the Biodex during all measures. The test was conducted with the arm at 90 degrees of abduction, 30 degrees of forward flexion, with the forearm supported in horizontal position to avoid the effects of gravity and the hand gripping a handle. The lever arm thus swivelled in the horizontal plane and its axis was substantially aligned with the axis of rotation of the elbow to allow free elbow flexion-extension movement. The amplitude of the movement was 60 degrees from a neutral position corresponding to an angle of 90 degrees at the elbow (30 degrees inward and 30 degrees outward). Maximal ECC task consisted of the participants trying forcefully to perform 5 consecutive extension movements (Figure 2) throughout the full range of motion while the dynamometer was motor driven from −30° to +30° and then relaxing for ~3–4 s while the forearm was returned passively to −30°. This was the inverse scenario for maximal CON task (5 consecutive flexion movements).

### 2.3. fNIRS Recordings

**Figure 3:**
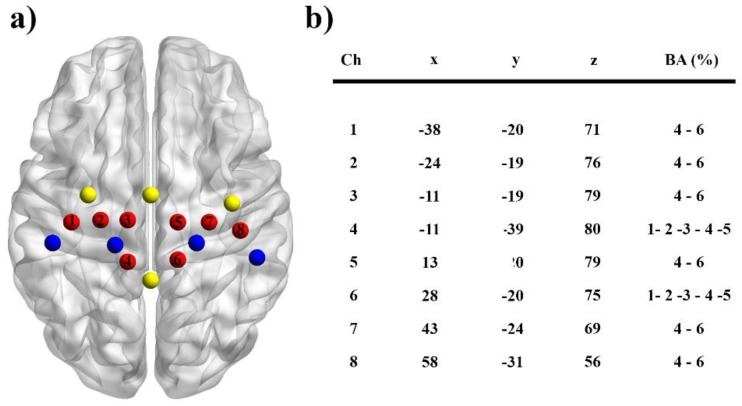
(**a**) Spatial organization of the fNIRS probes for the contralateral and ipsilateral primary motor (M1) cortices for one representative participant. Each M1 area was explored by 4 channels (in red) midway between the detectors (in blue) and the sources (in yellow) (BrainNet viewer toolbox). (**b**) MNI coordinates for each channel and corresponding Brodmann areas (BA).

Changes in O_2_Hb and HHb during rest and motor tasks were assessed using a continuous-wave NIRS system (Oxymon MkIII, Artinis Medical Systems, Einsteinweg, The Netherlands) utilizing two wavelengths (765 and 856 nm) at a sampling rate of 10 Hz. Before data collection the signal intensity was verified for each channel. During the recordings, the time course of changes in O_2_Hb and HHb concentration values were displayed in real time. A customized head cap with 16 NIRS channels from a 14-probe layout (4 detectors and 10 sources) was used to cover the motor cortex regions of the left and right hemispheres with a 3 cm source-detector distance (Figure 1a). Based on the international 10–20 EEG electrode system, the head cap with large ears holes and a chin belt was aligned with the Cz location of each participant. A 3-dimensional digitizer (Fastrak, Polhemus, Colchester, VT, USA) was used to measure the location of each fNIRS probe with a stylus marker in relation to four cranio-metrical landmarks of the participant’s head (nasion, Cz, the left and right pre-auricular points). Subsequently, these positional data were transformed to the Montreal Neurological Institute (MNI) coordinates system [16] and the points on the scalp were projected over a three-dimensional reconstruction of the brain cortex using the NIRS-SPM toolbox [17]. The Brodmann areas corresponding to the cortical regions were further determined using the Anatomy 1.8 toolbox for SPM [18]. Based on the anatomical locations, we designated two regions of interest (Figure 3b): contralateral and ipsilateral (as control) M1s to the movement. For that purpose, 8 channels with 8 optodes were retained for all subsequent analysis.

### 2.4. Data Analysis

#### 2.4.1. fNIRS Responses

After extracting the raw data (light intensity) from Oxysoft software (v3.0.95, Artinis Medical Systems), data were uploaded in Matlab^®^ (MATLAB 2014b, The MathWorks, ‎Natick, MA, USA) before converting them into optical density [19]. Pre-processing and artefacts removal were performed for each subject using Matlab scripts from the Homer toolbox (v2.2). The moving standard deviation and spline interpolation methods (SDThresh = 20, AMPThresh = 0.5, tMotion = 0.5 s, tMask = 2 s and *p* = 0.99) [20] were applied in combination with wavelet artefact correction (iqr = 0.1) [21] as recommended [22] in order to remove possible head motion artefacts. Then, the optical density data were converted to the relative concentrations (expressed in µM) of O_2_Hb and HHb using the modified Beer Lambert law that included an age-dependent constant differential path length factor [23]. Finally, a band pass filter zero-phase digital filter (4th order Butterworth, cut-off Frequency [0.08 0.009 Hz]) was applied to remove physiological noise like cardiac frequency, respiratory frequency, Mayer waves and very low frequencies [24]. We low pass filtered the fNIRS signals at 0.08 Hz since Mayer waves are low frequency oscillations that occur in human blood pressure signals at approximately 0.1 Hz. Plotting the power spectrum of each O_2_Hb time series helped us to assess the quality of the NIRS signals. The presence of a frequency peak of the cardiac activity around 1 Hz in the O_2_Hb signal indicates a good contact between the optical probe and the scalp [25]. Each channel that did not satisfy this criterion was discarded from further analysis. Following this step, 7 out of 16 participants were excluded due to insufficient NIRS signal quality.

The fNIRS time series in 9 participants were corrected to a zero baseline value at the onset of each set of the five repeated movements, which provided a reference value and allowed for standardized comparisons across each repetition and set. Relative changes of peak amplitude values of the O_2_Hb and HHb responses were extracted in a time interval of 5–15 s after motor task onset and averaged across the three sets of five movements for each experimental condition. Finally, the peak values for the four channels of each hemisphere covering M1 were averaged together resulting in an overall contralateral and ipsilateral response.

#### 2.4.2. Torque Values

The time series of the torque data collected directly from the dynamometer were extracted for calculations. Then, a homemade MATLAB script was used for determining the highest (peak) and standard deviation (SD) of the torque values across the duration of each elbow movement in a set for a given experimental condition. All values were averaged across the five movements and then the three sets for each experimental condition.

### 2.5. Statistical Analysis

Statistical analyses were performed using Statistica (V7.1, Statsoft Inc., Tulsa, OK, USA). Shapiro-Wilks test was used to identify if the data deviates from normal distribution. If not, a repeated measures ANOVA was used to compare peak values of O_2_Hb and HHb, peak and SD torque values with two within-subject factors (Movement: ECC and CON and velocity: 30°/s and 60°/s). Otherwise, two Wilcoxon rank-sum tests were used to assess the differences between the two modes of movement and the two angular velocities. Effect sizes were reported in the results section as follows: the partial-eta squared (η²) values [26] for the main and interaction effects of ANOVA when significant. Statistical differences were considered significant at *p* < 0.05. Data are reported as mean ± SD. 

## 3. Results

### 3.1. Torque Values

For the peak torque, there was no significant difference between ECC and CON movements (W = 67, *p* = 0.42, 38.02 ± 3.78 versus 39.91 ± 3.90 N m, respectively). However, torque values were significantly higher (W = 25, *p* = 0.0008) for velocity of 60°/s than that of 30°/s (41.18 ± 3.58 vs. 36.74 ± 4.11 N m, respectively). For the SD of torque, there was no effect for the movement × velocity interaction (F (1,7) = 2.06, *p* = 0.19) or the main effect of movement (F (1,7) = 0.13, *p* = 0.73) but SD values were significantly higher (F (1,7) = 14.6, *p* = 0.006, η² = 0.68) for velocity of 60°/s than that of 30°/s (12.11 ± 0.96 vs. 9.63 ± 0.98 N m, respectively). 

### 3.2. fNIRS Responses

**Figure 4:**
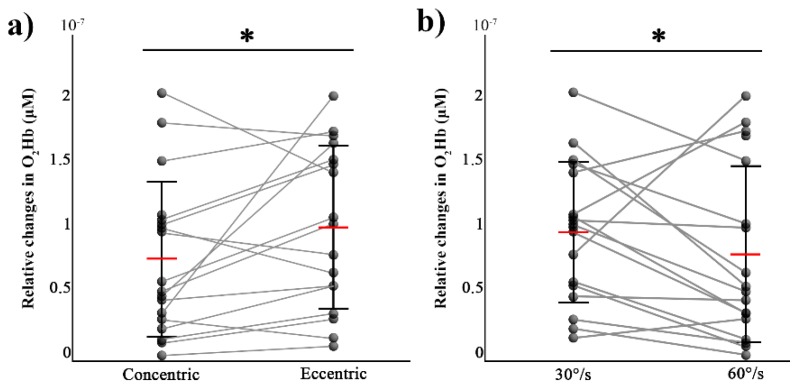
Changes for each participant (black circles) and mean (red horizontal line) values (± SD) of the peak values of O_2_Hb over the contralateral M1 during concentric and eccentric movements (**a**) and for two angular velocities ((**b**), 30°/s compared to 60°/s). * *p* < 0.05.

For the contralateral M1, there was a main effect of movement (F (1,7) = 6.3, *p* = 0.04, η² = 0.47) and a main effect of velocity (F (1,7) = 6.42 and *p* = 0.038, η² = 0.48) on O_2_Hb, with higher values in ECC and 60°/s conditions (see Figure 4). There was no significant effect for the movement × velocity interaction (F (1,7) = 0.055, *p* = 0.82). For HHb, there were no significant differences for the movement (W = 57, *p* = 0.36) and velocity (W = 53, *p* = 0.27).

For the ipsilateral M1, there were no significant differences on both O_2_Hb and HHb either for the movement (W = 69, *p* = 0.72; W = 62, *p* = 0.78) or for the velocity (W = 40, *p* = 0.08; W = 61, *p* = 0.71), respectively.

## 4. Discussion

The main objective of this study was to compare patterns of M1 activation between ECC and CON movements of elbow flexors at low-to-moderate angular velocities. The present findings provide further evidence that contralateral M1 activity to ECC movement was higher as compared to CON movement when maximally performed using an isokinetic ergometer. It was observed also that the slowest velocity of movement (30°/s) resulted in higher M1 activation than 60°/s regardless the mode of muscle actions. These results suggest a different central nervous system strategy to activate motor units when resisting an imposed load with regards to the angular velocities of movement investigated. 

During the present study, subjects were instructed to contract maximally over the range of movement (i.e., 60° of flexion or extension). Based on this typical isokinetic testing protocol [27], we are confident that the peak torque values (Figure 1) obtained for each movement was accurate within the context of the study. In addition, the velocity of movement was the same during ECC and CON conditions. Peak torque values independently of the type of movement decreased approximately 10% from 60°/s to 30°/s, suggesting an influence of slow to moderate angular velocities on peak torque values. For ECC movement, it can be explained by the shape of the torque-velocity relationship [28]. Regarding CON movement, an inverse scheme was expected, considering the assumed relationship between force versus velocity. With the forearm supported in horizontal position, we observed that peak torque values in the elbow flexors during CON conditions were roughly close at slow to moderate angular velocities. We attribute this finding to underlying potential biomechanical factors that require further investigations. The second finding related to the absence of differences in peak torque achieved during the two anisometric contractions was somewhat unexpected given the previous observations of Griffin [29] for elbow flexor muscles even if the differences are usually lower for slow angular velocities (<60°/s as investigated in the present study). This pattern might be affected by different levels of both voluntary activation and antagonist co-activation playing a significant role in determining the magnitude of the measured torque. However, the torque-EMG relationship of the elbow extensor muscles was not assessed in the present study for determining the level of antagonist co-activation. Additionally, the central activation ratio was not used for obtaining the voluntary activation level in the agonist muscle. The approach we adopted as a methodological strength of this study for the quantification of changes in brain activation at M1 during ECC and CON was to use the fNIRS method. The study of the human brain during functional tasks made a big step forwards with the introduction of non-invasive methods, among them the fNIRS [12,24]. Motor-task related changes in O_2_Hb and HHb over M1 areas measured by fNIRS reflect the hemodynamic response to neuronal activity, with good sensitivity [30]. However, physiological systemic noise arising notably from the cardiac activity and systemic blood pressure is a common component of signal interference in fNIRS measurements [24]. Even if we did not use reference channels to correct for superficial systemic interferences, attempts were made to reduce background physiological signals. We used a small muscle mass (elbow flexors) and methods dedicated to the analysis of fNIRS signals. Once optical signals had been checked and corrected for motion artefacts, we used band-pass filtering for removing many sources of physiological noise. In addition, cardiac activity responses were found comparable between ECC and CON for low metabolic loads [31]. In sum we assume that the observed changes in brain activation were likely not influenced by both systemic interferences and features of the movement (velocity and peak torque).

Literature still contains limited evidence that our central nervous system controls ECC movement differently than CON movements. These differences have been proposed at the level of the working muscle based on EMG signals. This is evidenced, for example, by the preferential recruitment of fast twitch motor units and different activation levels among synergistic muscles during ECC compared to CON muscle actions [32,33]. The level of agonist EMG activity under ECC is less than under CON isokinetic exercises at the same velocity [34]. At the cortical level, findings to date have been inconsistent regarding the specific activation patterns of M1. The present study showed that higher contralateral M1 activity by fNIRS occurred for ECC movements of elbow flexors at low-to-moderate angular velocities than for CON movements. These results are in agreement with previous observations from EEG studies [10,11] with similar paradigms but in contrast to a number of studies using fMRI measures of cortical activation with distinct experimental paradigms [6,8]. Using fMRI, ECC movements of human fingers [9,35] were found to result in stronger activation of M1. Nevertheless, Kwon and Park [6] found lower M1 in fMRI BOLD signal of the wrist extensors for a gradually increasing maximal effort without imposed loading. Imagined maximum ECC muscle actions of the elbow flexors as compared to CON condition also resulted in a decrease in M1 activity [8]. Neuroimaging methods are unlikely to be the main reason for this discrepancy between studies but rather the loading applied on the neuromuscular system at maximal exertion for manipulating the muscle action mode. In previous research, methods of obtaining submaximal and maximal muscle actions have differed (i.e., control the displacement of a load or a limb, or resist an imposed load) potentially confounding the comparability of the studies as a whole. In comparison with only one appropriate study [11], the present study used the isokinetic mode to maximally load the neuromuscular system through the overall range of motion. By controlling the mean angular velocity of movement we were able to provide more insight concerning the brain activity of M1 during both ECC and CON movements at maximal volitional effort. The result of the present study reinforces the first observation that the amplitude of the movement-related cortical potential derived from EEG over M1 was greater during maximal EEC than CON movements for the elbow flexors [11]. Our findings in contralateral M1 activity are consistent with those of Gruber et al. [36] who concluded that there is greater cortical excitability (i.e., increase in the motor evoked potentials-to-cervicomedullary evoked potentials ratio) during ECC compared with isometric contractions for the elbow flexor muscles. This indicates that properties of motoneurons as well as of neurons located at the motor cortex are likely modulated differently during ECC movements. Gruber et al. [36] suggested that increased cortical excitability would result in extra excitatory descending drive during muscle lengthening to compensate for spinal inhibition. 

Understanding the cortical encoding of muscle actions is of relevance to human function in the healthy state, as well as including people with neurological disorders and the responses to physical training or rehabilitation. We acknowledge that the generalization of our findings is limited due to the small sample size in this pilot study; the final number of subjects retained after analysis was 9 out of 16. Another potential limitation of the current study is that although we measured the cortical activity of M1, ECC or CON movements can act on more distant sites intra- and interhemispherically. Further studies could utilize more subjects to examine the reproducibility of these first findings and examine the interactions with other areas of the motor network through cortico-cortical connections [37] both intrahemispherically and across the corpus callosum.

## 5. Conclusions

The present study supports the muscle action mode-specificity concept on M1 activity by using the fNIRS signals associated with ECC movements as compared to CON movements. The main finding suggests that substantial cortical motor areas are activated differently during ECC movements at low-to-moderate angular velocities. Future research should, therefore, investigate open research questions on central nervous system strategies targeting ECC muscle action requirements related to functional motor performance in ecologically valid settings.
